# Independent Risk Factors for Urological Complications after Deceased Donor Kidney Transplantation

**DOI:** 10.1371/journal.pone.0091211

**Published:** 2014-03-07

**Authors:** Inez K. B. Slagt, Jan N. M. IJzermans, Laurents J. Visser, Willem Weimar, Joke I. Roodnat, Türkan Terkivatan

**Affiliations:** 1 Department of Surgery, Division of Transplant Surgery, Erasmus MC, University Medical Center, Rotterdam, the Netherlands; 2 Department of Internal Medicine, Division of Nephrology, Erasmus MC, University Medical Center, Rotterdam, the Netherlands; University of São Paulo State - Botucatu School of Medicine - UNESP, Brazil

## Abstract

Urological complications after kidney transplantation are mostly related to the ureteroneocystostomy, often requiring interventions with additional costs, morbidity and mortality. Our aim was to assess risk factors for urological complications in deceased donor kidney transplantation. Between January 2000 and December 2011, 566 kidney transplantations were performed with deceased donor kidneys. Recipients were divided in a group with, and a group without urological complications, defined as the need for a percutaneous nephrostomy catheter or surgical revision of the ureteroneocystostomy. Univariate and multivariate analyses were performed. Univariate analysis showed increased number of male donors (p = 0.041), male recipients (p = 0.002), pre-emptively transplanted recipients (p = 0.007), and arterial reconstructions (p = 0.004) in the group with urological complications. Less urological complications occurred in recipients on hemodialysis (p = 0.005). More overall surgical interventions (p<0.001), surgical site infections (p = 0.042), urinary tract infections (p<0.001) and lymphoceles (p<0.001) occurred in the group with urological complications. Multivariate analysis showed that male recipients (p = 0.010) and arterial reconstructions (p = 0.019) were independent risk factors. No difference was found between both groups in patient or graft survival. In conclusion, recipient male gender and arterial reconstruction are independent risk factors for urological complications after deceased donor kidney transplantation. Nevertheless, graft and recipient survival is not different between both groups.

## Introduction

Urological complications after kidney transplantation are reported to occur between 2.5% and 30% of all recipients [1], [5], [17], [21], [24]. Major urological complications, for example leakage and stenosis, are often related to the ureteroneocystostomy [2], [10], [13], [19], [22]. In most cases these complications require placement of a percutaneous nephrostomy (PCN). Sometimes, even a surgical revision is required, leading to additional morbidity and costs [4], [5], [22].

Risk factors that contribute to the prevalence of urological complications need to be determined. So far, many factors have been described in literature, including several donor and recipient characteristics [15], [21]. Furthermore, problems encountered during graft recovery, prolonged ischemia times, type of ureteroneocystostomy, presence of accessory arteries or stent placement might be of influence on the incidence of urological complications [2], [4], [20], [21].

Due to the increasing number of patients with end-stage kidney disease and a continuing shortage of donors, the demand for kidney grafts led to extension of donor criteria by the Dutch Transplant Foundation. Alongside the Donation after Brain Death (DBD) donors, Donation after Circulatory Death (DCD) (category III) donors have been deemed eligible for transplantation [9]–[12]. A higher percentage of urological complications after deceased kidney donation has been reported, when compared to live donor kidney transplantation [4], [22]. We aimed to assess the incidence of urological complications after kidney transplantation with grafts from DBD and DCD donors and identify independent factors associated with the development of these complications, in a multivariate analysis.

## Patients and Methods

The Erasmus MC, University Medical Center internal review board issued a formal written waiver for the need of ethics approval and the need for written informed consent.

Between January 2000 and December 2011, all kidney transplantations performed with grafts from DBD and DCD (category III) donors at the Erasmus University Medical Center Rotterdam, were reviewed retrospectively. A total of 566 recipients were identified. The surgical reports and electronic patient system were screened for donor and recipient characteristics, and urological complications. Recipients were divided in two groups, one group with and one group without urological complications within 3 months’ time after transplantation. A urological complication was defined as any event leading to the placement of a PCN or surgical revision of the ureteroneocystostomy during follow-up. We argued that a PCN placement is the best possible parameter to identify those patients who had an adverse urological outcome. An increasing serum creatinine level combined with hydronephrosis on ultrasonography was reason for a PCN placement. Monitoring of the PCN position and imaging of the ureter is performed by an antegrade pyelography (APG). If leakage of the ureteroneocystostomy is diagnosed with an APG, both PCN and urinary bladder catheter are placed until the leakage stops. In case the leakage is diagnosed shortly after transplantation immediate surgical reconstruction is performed. If a total obstruction of the ureter is diagnosed with an APG, surgical intervention is inevitable. If the APG shows a stenosed ureter but contrast reaches the bladder radiological dilation of the ureter is performed. Afterwards a percutaneous nephrocystostomy catheter (PCNC) is placed for 2 weeks. If the stenosis persists a surgical ureter reconstruction is indicated and will be performed by a transplant surgeon, together with an urologist.

### Overall Complications

Tacrolimus toxicity (>15 µg/l), suspected acute tubulus necrosis (ATN), treatment for rejection (methylprednisolone and/or ATG), lymphoceles, surgical site infections and urinary tract infections were scored during the first 3 months after transplantation. Besides ureteral revisions, all other re-interventions were documented: re-interventions because of re-bleeding, lymphocele drainage, transplantectomy and re-exploration because of vascular complications. Graft failure was defined as primary non-function or loss of function requiring dialysis. All recipients had a follow-up of at least one year in our center.

### Surgical Technique

All transplantations were performed by a transplant surgeon or vascular surgeon and transplants were engrafted extraperitoneally in the iliac fossa. In presence of multiple renal arteries (in majority of cases two arteries) a reconstruction was performed on the bench. Dependent on the length of the artery an end-to-side or an side-to-side anastomosis was created. Urinary continuity was established by either an intravesical [18] (Politano-Leadbetter) or extravesical [7] (Lich-Gregoir) ureteroneocystostomy. Intravesical anastomoses were created by performing a cystotomy on the anterior side to visualize the interior of the bladder and expose the trigone. A second (smaller) cystostomy was performed to create a new ureteric orifice. The ureter of the transplanted kidney as tunneled submucosally for approximately 2 centimeters. The distal end was trimmed, spatulated anteriorly at an optimal length to ensure a tension-free anastomosis and sutured to the bladder mucosa with 5–6 interrupted absorbable stitches. The cystotomy was then closed with a running suture. Extravesical anastomoses were created by performing a 1–2 centimeter cystotomy on the anterolateral surface of the bladder dome to expose the mucosa of the bladder wall. A small incision was made in the mucosa. The transplant ureter was trimmed and spatulated posteriorly. The mucosa of the bladder was sutured to the ureteral end with a running absorbable suture. The detrusor muscle was closed over the anastomosis using one or two interrupted absorbable sutures to create a submucosal tunnel with an anti-reflux mechanism. Placement of a stent depended on pre-transplant urinary production, so that urinary production of the transplanted kidney can be determined. Stents were externalized suprapubicly with the tip positioned in the pelvis of the graft and removed after 10 days.

### Postoperative Medical Care

Postoperatively, immunosuppressive therapy consisted of prednisolone (50 mg a day), tacrolimus (dose was titrated based on serum value) and mycophenolate mofetil (1000 mg twice a day). Basiliximab was used as induction therapy. Prednisolone was tapered and discontinued 4 months after transplantation. A prophylactic dose of 480 mg cotrimoxazole per day was given to prevent urinary tract infections. Cefazoline was given perioperatively. Standard dose of 12.000 U heparin daily was given during the first 5 post-operative day. Valganciclovir treatment was given to patients at risk for CMV infection or reactivation. Initial episodes of acute rejection were treated with methylprednisolone, 1000 mg a day for 3 days, ATG was given on indication.

### Statistical Analyses

Statistical analysis was performed using IBM SPSS version 20.0 (IBM Corp., Armonk, NY, USA). Variables studied are presented in Table 1 and Table 2. Categorical variables are presented as number (percentage) and were compared using the Chi-square test. Continuous variables are presented as mean with standard deviation (SD) and were compared using an independent sample T-test. We calculated odds ratios (OR) with 95% confidence intervals (CI), using a univariate and multivariate generalized linear model to identify independent risk factors for urological complications. All variables with a p-value <0.10 in the univariate analysis were included in the multivariate analysis. A p-value of <0.05 in our multivariate model was considered statistically significant. Graft and patient survival were analyzed using a Kaplan-Meier curve for survival distribution and compared using a log-rank test.

**Table 1:** Baseline characteristics of the donors, recipients and grafts (n = 566).

	Total group(n = 566)	No urological complication(n = 448)	With urological complication(n = 118)	p-value
**Donors**				
Male gender (%)	293 (51.8%)	222 (49.6%)	71 (60.2%)	0.041
Age (mean) (SD)	50.48 (14.45)	50.35 (14.28)	51.01 (15.12)	0.661
DBD (%)	352 (62.0%)	279 (62.3%)	73 (61.9%)	0.934
**Recipients**				
Male gender (%)	351 (62.0%)	263 (58.7%)	88 (74.6%)	0.002
Age (mean) (SD)	52.96 (13.95)	52.70 (13.77)	53.98 (14.65)	0.376
Multiple transplantations (%)	137 (24.2%)	108 (24.1%)	29 (24.6%)	0.916
Diabetes Mellitus (%)	130 (23.0%)	105 (23.4%)	25 (21.2%)	0.651
Pre-emptive transplantation (%)	20 (3.5%)	11 (2.5%)	9 (7.6%)	0.007
Hemodialysis (%)	383 (67.7%)	316 (70.5%)	67 (56.8%)	0.005
BMI (SD)	25.75 (5.03) (n = 516)	25.87 (5.11) (n = 405)	25.33 (4.75) (n = 111)	0.322
**Grafts**				
Warm ischemic time (mean in minutes) (SD)	38.67 (18.72)	38.76 (19.42)	38.36 (15.89)	0.837
Cold ischemic time (mean in minutes) (SD)	1083.42 (350.59)	1083.57 (349.37)	1082.84 (356.73)	0.984
Arterial reconstruction (%)	70 (12.4%)	46 (10.3%)	24 (20.3%)	0.004
Extravesical ureteroneocystostomy (%)	124 (21.9%)	98 (22.1%)	26 (22.4%)	0.946
Stent placement (%)	273 (48.2%)	213 (47.7%)	60 (51.3%)	0.484

*p-value is provided between the group without urological complications and the group with urological complications.

SD: Standard Deviation; DBD: Donation after Brain Death; BMI: Body Mass Index.

**Table 2:** Overall complications.

	Total Group(n = 566)	No urological complication(n = 448)	With urological Complication(n = 118)	p-value*
Overall surgical intervention (%)	132 (23.3%)	85 (19.0%)	47 (39.8%)	<0.001
ATN (%)	240 (42.4%)	187 (41.7%)	53 (44.9%)	0.535
Tacrolimus toxicity (>15 µg/l) (%)	63 (11.1%)	47 (10.5%)	16 (13.6%)	0.346
Surgical site infection (%)	50 (8.8%)	34 (7.6%)	16 (13.6%)	0.042
Urinary tract infection (%)	130 (23.0%)	84 (18.8%)	46 (39.0%)	<0.001
Lymphocele (%)	17 (3.0%)	3 (0.7%)	14 (11.9%)	<0.001
Rejection treatment (%)	71 (12.5%)	54 (12.0%)	17 (14.4%)	0.492
Primary non-function (%)	51 (9.0%)	46 (10.3%)	5 (4.2%)	0.042

*p-value is provided between the group without urological complications and the group with urological complications.

PCN: Percutaneous Nephrostomy; ATN: Acute Tubulus Necrosis.

### Ethics

The manuscript is conducted in accordance to the principles expressed in the Declaration of Helsinki. Approval of our local ethics committee was not required for this study.

## Results

### Baseline Characteristics

Between January 2000 and December 2011, 566 kidney transplantations were performed with grafts from both DBD and DCD donors. An overview of the baseline characteristics is shown in Table 1. Urological complications were significantly more frequent in male donors, male recipients, pre-emptive transplantations and arterial reconstructions. Significantly less recipients on hemodialysis developed a urological complication. The number of kidney grafts from DBD and DCD donors was equally distributed in the group with and without urological complications.

### Urological Complications

Of the total 566 recipients, 117 received a PCN. In 15 recipients a PCN was placed because of leakage and in 102 because of hydronephrosis on ultrasonography. An endoscopic dilatation of the ureter was performed in 4 recipients, in 3 patients successfully and in 1 recipient an additional surgical revision was required afterwards. A surgical ureteral revision was required in 31 recipients who previously received a PCN and in one recipient a surgical ureteral revision was required without a prior PCN placement based on leakage of the ureter shortly after transplantation. Choice of re-implantation was in 30 cases a new ureterovesicostomy, in one patient a pyelovesicostmy and in one a ureter-ureterostomy. Mean graft survival of the group with a surgical ureter reconstruction was 5.57 years (inter quartile range of 2.14–9.27). In 83 recipients, the PCN could be removed without additional intervention.

### Overall Complications

Comparisons of the overall complications according to absence or presence of urological complications are presented in Table 2. There were significantly more overall surgical interventions, surgical site infections, urinary tract infections and lymphoceles in the group of recipients with urological complications. Primary non-function prevailed significantly less frequently in recipients with urological complications.

### Multivariate Analysis

All odds ratios regarding urological complications using univariate and multivariate analysis were presented in Table 3. Five factors (donor gender, recipient gender, pre-emptive transplantation, hemodialysis, arterial reconstruction) implemented in our univariate analysis showed a significant influence on the presence of urological complications and were therefore analyzed in a multivariate model. Recipient gender and arterial reconstruction were identified as independent risk factors in our multivariate analysis.

**Table 3:** Results of the multivariate analysis regarding urological complications.

Variable	Univariate OR (95% CI)	p-value*	Multivariate OR (95% CI)	p-value*
**Donors**				
Male gender	1.57 (1.02–2.33)	0.041	1.46 (0.96–2.24)	0.080
**Recipients**				
Male gender	2.06 (1.32–3.29)	0.002	1.84 (1.15–2.93)	0.010
Pre-emptive transplantation	3.28 (1.29–8.12)	0.007	2.20 (0.82–5.81)	0.111
Hemodialysis	0.55 (0.36–0.83)	0.005	0.66 (0.42–1.04)	0.073
**Grafts**				
Arterial reconstruction	2.23 (1.28–3.81)	0.004	1.96 (1.10–3.40)	0.019

*p-value is provided between the group without urological complications and the group with urological complications.

OR: Odds Ratio; CI: Confidence Interval.

### Donor Type and Urological Complications

In total 118 (20.8%) recipients developed a urological complication and 5.7% of all recipients (32 out of 566) underwent a surgical ureteral revision. Of all recipients who developed a urological complication, 73 had a DBD donor graft and 45 a DCD. Table 4 shows that DBD and DCD transplantations were not different regarding prevalence of urological complications. However, there were significantly more surgical site infections in the DCD group.

**Table 4:** Characteristics and complications of the recipients with urological complications.

	Total (n = 118)	DBD (n = 73)	DCD (n = 45)	p-value*
Ureteral reconstruction (%)	32 (27.1%)	24 (32.9%)	8 (17.8%)	0.130
Male gender recipient (%)	88 (74.6%)	54 (74.0%)	34 (75.6%)	0.848
Male gender donor (%)	71 (60.2%)	41 (56.2%)	30 (66.7%)	0.258
Arterial reconstruction (%)	24 (20.3%)	16 (21.9%)	8 (17.8%)	0.587
Urinary tract infection (%)	46 (39.0%)	28 (38.4%)	18 (40.0%)	0.859
Surgical site infection (%)	16 (13.6%)	6 (8.2%)	10 (22.2%)	0.031
Lymphocele (%)	14 (11.9%)	9 (12.3%)	5 (11.1%)	0.843

*p-value is provided between the DBD and DCD group.

DBD: Donation after Brain Death; DCD: Donation after Circulatory Death;

PCN: Percutaneous Nephrostomy.

### Follow-up

**Figure 1:**
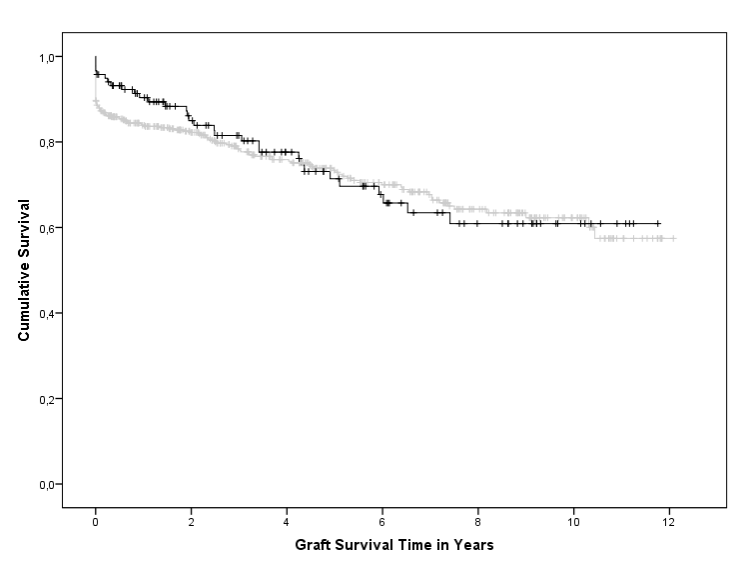
Kaplan-Meier survival, the black line corresponds to the group with urological complications and the grey line corresponds to the group without urological complications. This curve is censored for death. No significant difference occurred between both groups (p = 0.707).

Mean graft survival time was 4.02 years with a standard deviation of 3.47. Minimum graft survival was 0 day due to primary non function and maximum was 12.1 years. Mean patient survival was 5.13 years. Death censored graft survival regarding urological complications was presented by a Kaplan-Meier curve (Figure 1). No significant difference occurred in graft survival between the group with or without urological complications (p = 0.707).

## Discussion

In our study, 20.8% of all recipients of a kidney graft from a deceased donor had a urological complication as defined by PCN placement or surgical ureteral revision. As PCN placement is considered as a minimally invasive event in our center, the threshold to use a PCN is low, either for therapy, or for diagnosis. Eventually, only 32 recipients (5.7%) underwent a surgical ureteral revision for leakage or stenosis and graft survival was not worse in the population with urological complications.

It has been suggested that urological complications are caused by an insufficient blood supply to the ureter. Excessive dissection of the site known as ‘golden triangle’ (the site confined by ureter, kidney and renal artery) should therefore be avoided during graft recovery. Damage of this triangle might lead to necrosis of the distal ureter in 70% of the cases [4], [13], [17].

Potential risk factors for urological complications including age, prolonged cold ischemia and recipient Diabetes Mellitus were reported not to play an important role in the occurrence of urological complications [4], [21]. These findings are supported by our data (Table 1). In our study more urological complications occurred in male donors, male recipients and pre-emptive transplantations. The reason why male recipients may develop more urological complications is not exactly clear. An anatomical cause due to the presence of the funiculus that crosses the ureter might be an explanation, considering the ligamentum rotundum is ligated in females during the implantation. Furthermore, arterial reconstruction of the donor graft was highly associated with the prevalence of urological complications (Table 1), which is confirmed in the literature [3], [6], [14]. Relative ischemia of the ureter by an insufficient arterial blood supply is suggested to be the cause for leakage and stenosis. Malperfusion of accessory arteries may result from a small anastomosis with flow-limitation, greater turbulence or more vulnerability for traction injury.

There is growing evidence on the superiority of the extravesical ureteroneocystostomy when compared with the intravesical technique, with or without additional routine stent placement [1], [2], [8], [16], [20], [23]. In our population, the type of ureteroneocystostomy was depended on surgeons’ preference and stent placement on residual urinary production. Both, type of ureteroneocystostomy and the presence of a stent could not be defined as a risk factor in our analysis (Table 1). However, there were significantly more surgical site infections, urinary tract infections, lymphoceles and surgical re-explorations because of hemorrhage, transplantectomies or vascular complications (Table 2), in the group with urological complications.

Although the mean graft survival of DCD donors is suspected to be shorter than that of DBD donors, there are no studies on the occurrence of urological complications in those groups. Therefore, the finding that grafts from a DBD donor were not superior to DCD donor grafts with respect to urological complications (Table 4) is an important finding. In addition, our Kaplan Meier survival analysis (Figure 1) demonstrates no difference in long term graft survival between the populations with and without urological complications which is supported by other studies [1], [4], [22]. It should however be kept in mind that the population with urological complications is a selection with a functioning graft.

Another important finding in our study was the fact that primary non-function of the graft or graft loss within 3 months was significantly lower in the group without urological complications (Table 2). This probably is a bias since urological complications like leakage and hydronephrosis by a stenosis at the ureteroneocystostomy junction site cannot be detected in a non-functioning graft. Furthermore, recipients transplanted pre-emptively, had a significantly higher risk to develop urological complications. There is no clear explanation for this finding. However, this subgroup consists of only 20 recipients, which might have biased the statistical outcome. One other limitation of our study is the fact that some potential risk factors, such as donor BMI, ureteral vascularization or length of the ureter could not be documented prospectively. Despite the retrospective character of our study and its disadvantages, we describe the most detailed group regarding urological complications of kidney graft recipients from a deceased donor so far as known from the literature.

Based on our study of kidney transplantations from a deceased donor, we conclude that recipient’s gender and arterial reconstruction are independent risk factors to develop a urological complication. However, donor type (DBD and DCD), primary non-function, type of anastomosis, and the presence of multiple transplantations could not be defined as risk factor in our univariate and multivariate analyses. This means that donor type and surgical anastomosis technique are less important factors for the urological outcome, which is in contrast to what one might argue.
